# Direct Reprogramming Rather than iPSC-Based Reprogramming Maintains Aging Hallmarks in Human Motor Neurons

**DOI:** 10.3389/fnmol.2017.00359

**Published:** 2017-11-02

**Authors:** Yu Tang, Meng-Lu Liu, Tong Zang, Chun-Li Zhang

**Affiliations:** ^1^Department of Molecular Biology, University of Texas Southwestern Medical Center, Dallas, TX, United States; ^2^Hamon Center for Regenerative Science and Medicine, University of Texas Southwestern Medical Center, Dallas, TX, United States; ^3^National Clinical Research Center for Geriatric Disorders, Xiangya Hospital of Central South University, Changsha, China

**Keywords:** human iPSC, neural progenitor cells, motor neuron, ALS, direct reprogramming, aging, rejuvenation

## Abstract

*In vitro* generation of motor neurons (MNs) is a promising approach for modeling motor neuron diseases (MNDs) such as amyotrophic lateral sclerosis (ALS). As aging is a leading risk factor for the development of neurodegeneration, it is important to recapitulate age-related characteristics by using MNs at pathogenic ages. So far, cell reprogramming through induced pluripotent stem cells (iPSCs) and direct reprogramming from primary fibroblasts are two major strategies to obtain populations of MNs. While iPSC generation must go across the epigenetic landscape toward the pluripotent state, directly converted MNs might have the advantage of preserving aging-associated features from fibroblast donors. In this study, we confirmed that human iPSCs reset the aging status derived from their old donors, such as telomere attrition and cellular senescence. We then applied a set of transcription factors to induce MNs from either primary fibroblasts or iPSC-derived neural progenitor cells. The results revealed that directly reprogrammed MNs, rather than iPSC-derived MNs, maintained the aging hallmarks of old donors, including extensive DNA damage, loss of heterochromatin and nuclear organization, and increased SA-β-Gal activity. iPSC-derived MNs did not regain those aging memories from old donors. Collectively, our study indicates rejuvenation in the iPSC-based model, as well as aging maintenance in direct reprogramming of MNs. As such, the directly reprogrammed MNs may be more suitable for modeling the late-onset pathogenesis of MNDs.

## Introduction

Motor neuron diseases (MNDs) are a heterogeneous group of neurologic disorders characterized by the selective loss of motor neurons (MNs) in spinal cord and motor cortex (Robberecht and Philips, 2013; Winner et al., 2014), including amyotrophic lateral sclerosis (ALS) and spinal muscular atrophy (SMA). Since it is rarely possible to obtain diseased cells directly from patients, *in vitro* generation of MNs becomes an attractive approach for modeling and potentially treating MNDs. So far, two major cell reprogramming strategies have been applied to generate MNs. The usage of human induced pluripotent stem cells (iPSCs) derived from primary fibroblasts has opened the new era of cell reprogramming (Takahashi et al., 2007), and helped to obtain ample cell samples for *in vitro* studies on neurodegenerative diseases. Specifically, iPSCs from patients with ALS harbor the patient’s complex genetic makeup, and can be subsequently differentiated toward MNs, albeit with varied conversion efficiencies (Chen et al., 2014; Kiskinis et al., 2014). Alternatively, neurons can be directly reprogrammed *in vitro* from primary fibroblasts by introducing specific transcription factors or microRNAs (Masserdotti et al., 2016). By virtue of different sets of induction factors, different subtypes of neurons including mixed neurons (Vierbuchen et al., 2010; Pang et al., 2011; Ladewig et al., 2012), dopaminergic neurons (DAs) (Caiazzo et al., 2011; Pfisterer et al., 2011; Jiang et al., 2015), and medium spiny neurons (Victor et al., 2014; Richner et al., 2015) have been successfully obtained in a dish. Previous work in our lab has also established a mature platform to generate directly induced MNs from patient fibroblasts (Fib-iMNs). We employed four transcription factors including NGN2, SOX11, ISL1, and LHX3 in combination with a cocktail of small molecules to yield functional Fib-iMNs from both human fetal and adult fibroblasts with high efficiencies (Liu et al., 2013, 2016).

The iPSC-based model has provided profound insights into the pathogenesis mechanisms, and drug screening evaluation in functional neurons in culture (Yang et al., 2013; Sterneckert et al., 2014). However, generally differentiated neurons from iPSCs are known to be immature that often require long-term culture to develop mature functional properties (Kriks et al., 2011; Sances et al., 2016). Moreover, numerous studies also reported that iPSC-derived neurons only recapitulate early biochemical phenotypes without mimicking the late-onset severe neurodegenerative hallmarks of the disease (Nguyen et al., 2011; Seibler et al., 2011; Patterson et al., 2012). This may be due to a lack of the preservation of aging status, since cell reprogramming must transit through an embryo-like pluripotent state accompanied by a loss of particular age-associated features (Lapasset et al., 2011).

Aging is by far the most critical risk factor in many forms of late-onset neurodegenerative disorders. Specifically in ALS, the risk increases with age, and the onset of symptoms usually occurs in midlife between the ages of 45 and 60, although the disease can also occur earlier (Boillee et al., 2006). At cellular levels, progressive aging leads to declines in physiology and functionality of adult tissues in many aspects (Lopez-Otin et al., 2013), including declines in neuronal plasticity and cognitive performances (Burke and Barnes, 2006) and accumulation of damaged DNA and proteins in neurons (Mattson and Magnus, 2006). Apart from genetic susceptibility, cellular aging process definitely takes pivotal roles in the development of MNDs. Thus, it is particularly important to recapitulate age-related characteristics by using neurons that reflect the age at which the disease develops.

Direct conversion of neurons from fibroblasts circumvents the pluripotent stage and therefore may preserve the aging memories and render the converted neurons to be age-equivalent with their donors. Consistent with this idea, emerging data showed that directly converted brain neurons retain certain aging-associated features (Mertens et al., 2015; Huh et al., 2016). However, no study has examined the aging status of induced spinal cord neurons such as Fib-iMNs.

In this study, we first confirmed that rejuvenation occurs in human iPSCs that are generated even from old donors. We then used the same procedure to induce MNs either directly from human fibroblast donors or from iPSC-derived neural progenitor cells (NPCs). A spectrum of aging-associated markers, such as nuclear morphology, nuclear organization, DNA damage, heterochromatin and senescence-activated β-galactosidase (SA-β-Gal) activity, were then used to evaluate the aging status between Fib-iMNs and iPSC-derived MNs (iPSC-MNs). Our results indicate that age-associated features are maintained in directly converted Fib-iMNs but not iPSC-MNs, suggesting that Fib-iMNs may be better suited to studying late-onset pathogenesis of MNDs.

## Materials and Methods

### Cell Culture

All human primary fibroblasts and some human iPSC lines were obtained from the commercial Coriell Cell Repository (**Table 1**). iPS-CHOPWT1.1 and iPS-CHOPWT2.2 lines were kindly provided by Dr. Paul Gadue (Mills et al., 2013). Primary fibroblasts were maintained in fibroblast medium: DMEM medium supplemented with 15% fetal bovine serum (FBS, Corning, NY, United States), and 1% penicillin/streptomycin (P/S) at 37°C and 5% CO_2_. Human iPSCs were cultured in complete mTeSR1 medium (STEMCELL Technologies, Vancouver, BC, Canada) on Matrigel (Corning) coated dishes at 37°C and 5% CO_2_, and the medium was daily replaced.

**Table 1:** List of human primary fibroblasts and iPSC lines.

Status	ID	Fibroblast	iPSC	Description	Age	Race	Gender	Affected	Gene	Mutation
**Young**	Y1	GM00041	GM23411	Normal	3 Months	Caucasian	F	No		
	Y2	GM00969	CHOPWT1.1	Normal	2 years	Caucasian	F	No		
	Y3	GM05565	CHOPWT2.2	Normal	3 years	Hispanic	M	No		
**Old**	O1	AG09969	Self-Made	Normal	53 years	Caucasian	M	No		
	O2	GM23248	GM23338	Normal	55 years	Caucasian	M	No		
	O3	AG05811	Self-Made	Normal	71 years	Caucasian	F	No		
**Diseased**	ALS1		ND35660	ALS1	50 years	Caucasian	F	Yes	SOD1	G90A
	ALS2		ND35664	ALS1	68 years	Caucasian	F	Yes	SOD1	G90A
	ALS3		ND39034	ALS1	37 years	Caucasian	M	Yes	FUS	c.1566G>A (p.522R)
	ALS4		ND35663	ALS1	50 years	African American	F	Yes	FUS	g.11194C>G (p.H517Q)

The medium recipes were as the following:

(1) Embryonic stem cell (ESC) medium: DMEM/F12 medium with 20% KnockOut Serum Replacement (KSR, Thermo Fisher Scientific, MA, United States), 1% GlutaMax, 1% non-essential amino acid (NEAA), 50 μM β-mercaptoethanol (β-ME), 1% P/S and 10 ng/ml basic fibroblast growth factor (bFGF, PeproTech, Rocky Hill, NJ, United States).
(2) KSR medium: DMEM/F12 medium with 20% KSR, 1% GlutaMax, 1% NEAA, 50 μM β-ME, and 1% P/S.
(3) Neurosphere medium (NSP medium): DMEM/F12 medium containing 1% N2, 1% GlutaMax, 1% NEAA, 50 μM β-ME, 1% P/S, 8 μg/ml Heparin, 20 ng/ml bFGF, and 20 ng/ml epidermal growth factor (EGF, PeproTech).
(4) NPC medium: DMEM/F12 and neurobasal medium (1:1) containing 0.5% N2 (Invitrogen, Carlsbad, CA, United States), 1% B27 (Invitrogen), 1% GlutaMax, 1% NEAA, 50 μM β-ME, 1% P/S, 10 ng/ml EGF, and 10 ng/ml bFGF.
(5) C2 medium: DMEM/F12 and neurobasal medium (2:1) containing 0.8% N2, 0.4% B27, and 1% P/S. C2 medium was supplemented with 5 μM forskolin (FSK, Sigma, St. Louis, MO, United States) and 10 ng/ml each of BDNF, GDNF, and NT3 (PeproTech) for neuron culture.

### Virus Preparation

For retrovirus, four moloney-based retroviral vectors (pMXs) carrying human complementary DNAs (cDNAs) of OCT4, SOX2, KLF4, and c-MYC were obtained from Addgene (Takahashi et al., 2007). Each of these plasmids was co-transfected with two packaging vectors (pCMV-Gag-Pol and pCMV-VSVG) into HEK293T cells using polyethylenimine (PEI) reagent. For lentivirus, the third-generation replication-deficient lentiviral vector (pCSC-SP-PW-IRES-GFP) was used to express NGN2-IRES-GFP-T2A-mSOX11 and ISL1-T2A-LHX3. Lentiviruses were then generated by co-transfections of HEK293T cells with lentiviral vectors together with three packaging plasmids (pMDL, VSV-G, and pREV). Viral supernatants were collected 48 and 72 h post-transfection and filtered by 0.45 μm syringe filters.

### iPSC Generation

Human primary fibroblasts were plated at 8 × 10^5^ cells/dish onto gelatin-coated 10-cm dishes, and were infected by the cocktail of four retroviruses (1:1:1:1) at five MOI expressing OCT4, SOX2, KLF4, and c-MYC. Two days later, fibroblasts were received a second round of virus infection, and this time point was marked as day 0. Fibroblasts were then replated at day 5 in fibroblast medium typically at 2 × 10^5^ cells per well in Matrigel-coated 6-well plates. Cells were cultured with human ESC medium at day 6, which was gradually replaced by mTeSR1 medium in the following few days. Treatment with 0.5 mM VPA (Sigma) begins at day 5 and lasts for 10 days. To establish iPSC lines, colonies were picked around 3 weeks to 1 month post-infection based on ESC-like colony morphology. The picked colonies were then expanded and maintained on Matrigel-coated plates with mTeSR1 medium for feeder-free culture until characterization.

### Embryoid Bodies (EBs)

For EBs formation, human iPSCs were dissociated by Versene (Thermo Fisher Scientific) treatment and transferred to low-attachment 10-cm petri dishes (Grainger, Peoria, IL, United States) in KSR medium supplemented with 10 μM ROCK inhibitor Y-27632 (STEMCELL Technologies). The medium was replaced every other day. After 7 days in suspension culture, EBs were digested with 0.25% trypsin and cultured on gelatin-coated plates with KSR medium for another 7 days.

### NPC Generation and Differentiation

Neural progenitor cell generation was performed according to a previously reported protocol with minor modifications (Yang et al., 2014). Briefly, a total 2 × 10^6^ undifferentiated iPSCs were split by Versene into three wells of a Matrigel-coated 6-well plate, and were then cultured in mTeSR1 medium with 10 μM all-*trans*-retinoic acid (RA, Sigma) and 0.5 mM VPA for 7 days. Those cells were then digested with Versene and gently pipetted into small clumps supplemented with 10 μM Y-27632 in low-attachment 10-cm Grainger petri dishes. The cell clumps were aggregated in KSR medium for 4 days to form neurospheres, followed by culturing in NSP medium for another 1 week. Neurospheres were then dissociated into single cells by accutase (Innovative Cell Technologies, San Diego, CA, United States), and finally maintained in NPC medium.

For spontaneous differentiation, NPCs were plated onto Matrigel-coated plates at a density of 3 × 10^4^ cells/cm^2^ and cultured with C2 medium containing 5 μM FSK and 10 ng/ml each of BDNF, GDNF, and NT3. The medium was half-changed every other day until analysis. For MN differentiation with high efficiency, NPCs were seeded onto Matrigel-coated plates at a density of 3 × 10^4^ cells/cm^2^ for overnight and infected with a cocktail of two lentiviruses (1:1) at 50 MOI expressing NGN2-IRES-GFP-T2A-mSOX11 and ISL1-T2A-LHX3, respectively, in the presence of 6 μg/ml polybrene. The next day, the medium was replaced with C2 medium containing 5 μM FSK and 10 ng/ml each of BDNF, GDNF, and NT3. Neurons were then dissociated by accutase at 6 days post infection (dpi) and replated onto astrocyte-coated coverslips at a density of 1 × 10^3^ cells/cm^2^. The medium was changed twice a week until analysis.

### Direct Reprogramming

Direct reprogramming was conducted according to our previous protocol (Liu et al., 2016). In brief, fibroblasts were digested with 0.25% Trypsin at 37°C and plated at 8 × 10^5^ cells/dish onto Matrigel-coated 10-cm culture dishes. Cells were infected the next day with a cocktail of two lentiviruses (1:1) at 100 MOI expressing NGN2-IRES-GFP-T2A-mSOX11 and ISL1-T2A-LHX3, supplemented with 6 μg/ml polybrene. Fibroblast medium was refreshed after overnight incubation. One day later, the culture was switched to C2 medium containing 10 μM FSK, 1 μM dorsomorphin (DM, Millipore, MA, United States) and 10 ng/ml bFGF. The supplemented C2 medium was half-changed every other day. Typical neuron morphology generally emerged after 6 dpi. To purify the induced neurons from non-converted fibroblasts, we employed a procedure of differential plating. Briefly, cells at 14 dpi were rinsed with Dulbecco’s phosphate-buffered saline, digested with 0.025% trypsin at 37°C for 15 min, and resuspended with fibroblast medium to quench trypsin activity. The cell suspension was then transferred onto a gelatin-coated 10-cm dish to which fibroblasts tightly attached. Around 30 min later, floating cells, which were mainly induced neurons, were harvested by centrifugation at 500 × *g* for 2 min. Cell pellets were resuspended with C2 medium supplemented with 5 μM FSK and 10 ng/ml each of BDNF, GDNF, and NT3 (PeproTech), and were finally plated onto astrocyte-coated coverslips. Unless indicated otherwise, C2 medium with neurotrophic factors were half-changed twice a week until analysis.

### Neuron Survival

Motor neurons derived from wild type and diseased iPSCs were replated onto astrocyte-coated coverslips for survival assessment. Primary astrocytes were isolated from postnatal day 1 (P1) mouse pups according to previously described procedures (Tang et al., 2014). Endogenous mouse neurons were removed by a few cycles of passaging, freezing, thawing, and plating. Those iPSC-MNs at 11 dpi were replated onto astrocyte-coated 96-well plates. A similar number of starting cells were plated to facilitate calculating the survival rates. Culture medium was half-changed twice a week with C2 medium containing 5 μM FSK. Cells were fixed and immunostained for Tuj1 and GFP at 14 dpi, 21 dpi, and 35 dpi. Tuj1^+^GFP^+^ cells within the entire well of a 96-well plate in triplicate were quantified under an AMG EVOS digital inverted fluorescence microscope. Cell counts were normalized to the starting number of cells in each well.

### Alkaline Phosphatase (AP) Staining and Karyotyping

Alkaline phosphatase staining was performed with the Vector Blue Substrate kit (SK-5300) from Vector Laboratories. Briefly, iPSCs were fixed with 4% paraformaldehyde (PFA) supplemented with 0.1% Triton X-100 for 10 min. After rinses with PBS and H_2_O, cells were then incubated with freshly prepared AP working solution in the dark for 40 min. Karyotype analysis of iPSCs was performed at WiCell Research Institute.

### SA-β-Gal and Mitochondrial Superoxide

Cell senescence was analyzed using a Senescence-β-Galactosidase Staining Kit (#9860, Cell Signaling). Briefly, cells were washed twice with PBS and fixed with 1x fixative solution for 15 min at room temperature. After rinsing twice with PBS, cells were incubated with 1x β-galactosidase staining solution at 37°C without CO_2_. Cells were finally counterstained with Hoechst 33342. Mitochondrial superoxide was detected using a MitoSOX^TM^ Red mitochondrial superoxide indicator (M36008, Molecular Probes). Briefly, cells were incubated with 5 μM MitoSOX^TM^ reagent working solution for 10 min at 37°C, protected from light. After gently washing for three times with warm PBS, cells were counterstained with Hoechst 33342 before observation under a fluorescent microscope.

### Bisulfite Sequencing

Genomic DNA from fibroblasts and iPSCs were isolated with lysis buffer containing 0.5 mg/ml proteinase K. Bisulfite conversion of genomic DNA was carried out using the EZ DNA Methylation-Direct Kit (Zymo Research) according to the manufacturer’s instructions. A genomic fragment of the OCT4 promoter was amplified using EpiMark Taq DNA polymerase (New England Biolabs). Primer sequences were previously published (Liu et al., 2011) and listed in Supplementary File 1. PCR products were then purified by gel extraction using Zymoclean Gel DNA Recovery Kit (Zymo Research), and subsequently cloned into the pMD20-T vector (Takara, Shiga, Japan). Clones were picked from each sample and sequenced by the M13Rev universal primer.

### Telo-PCR

Telo-PCR was performed according to a previously published protocol with some modifications (Cawthon, 2002). This assay determines a relative telomere length in telomeric DNA by measuring the T/S ratio of telomere repeat copy number (T) to singe gene copy number (S). The single gene termed acidic ribosomal phosphoprotein PO (36B4) was used as the endogenous control. Primer sequences were listed in Supplementary File 1.

### Gene Expression

Total RNAs were extracted from cultured cells using TRIzol reagents (Invitrogen) and was reverse-transcripted into cDNA using a SuperScript III kit (Invitrogen). Quantitative real-time PCR (qRT-PCR) was then performed using SYBR GreenER SuperMix (Invitrogen) and carried out on a 384-well ABI 7900HT thermocycler (Applied Biosystems). The PCR program was consisted of 2 min at 50°C and 10 min at 95°C, followed by 40 cycles of 15 s at 95°C and 45 s at 62°C. All primers were verified by melting curve analysis containing a single melt curve peak. Primer sequences were listed in Supplementary File 1. The relative expression level of each gene was analyzed using the 2^-ΔΔC_t_^ algorithm normalized to the housekeeping gene hypoxanthine guanine phosphoribosyltransferase (HPRT) and relative to control samples.

### Immunocytochemistry and Confocal Microscopy

Cultured cells were fixed with 4% PFA for 15 min at room temperature. After a brief rinse with PBS, cells were incubated with blocking buffer (PBS containing 0.2% Triton X-100 and 3% BSA) for 30 min at room temperature, followed by an overnight incubation at 4°C with the following primary antibodies: OCT4 (1:50; Santa Cruz sc-5279), SOX2 (1:50; Santa Cruz sc-17320), SSEA4 (1:1000; DSHB MC-813-70), Lamin B1 (1:500; PeproTech 12987-1-AP), γH2AX (1:500; Millipore #05-636), H3K9me3 (1:5000; Abcam ab8898), LAP2α (1:100; Abcam ab5162), HP1γ (1:200; Cell Signaling #2619), PSA-NCAM (1:250; DSHB #5A5), Ki67 (1:500; Leica Biosystems NCL-Ki67p), PAX6 (1:500; Sigma HPA030775), NESTIN (1:500; Millipore MAB5326), Tuj1 (1:2000; Covance PRB-435P), Tuj1 (1:2000; Covance MMS-435P), MAP2 (1:500; Sigma M4403), GFP (1:1000; Aves GFP-1020); GFAP (1:500; Sigma G3893), ISL1 (1:100; DSHB 39.3F7), HB9 (1:100; DSHB 81.5C10-c), ChAT (1:200; Millipore AB144P), TH (1:1000; Aves #TYH), GABA (1:1000; Sigma A2052), vGlut1 (1:100; UC Davis 75-066), vGlut3 (1:50; UC Davis 75-073), and Synapsin (1:200; Cell Signaling #5297). After repeated washes, cells were incubated with Alexa Fluor-conjugated secondary antibodies made in donkey (Invitrogen, 1:500) at room temperature for 1 h. The nuclei were finally stained with Hoechst 33342. Confocal images were obtained with a Nikon A1R Confocal Microscope. Fluorescence intensity was quantified using ImageJ with a plugin of ND to Image6D. Relative fluorescence intensity (RFI) of aging markers was measured only in the nuclei, and normalized to Hoechst intensity.

### Electrophysiology

Reprogrammed MNs were cultured on astrocyte-coated coverslips for 35 days and used for analysis. Cells were maintained at 30°C in a submersion chamber with Tyrode solution containing 150 mM NaCl, 4 mM KCl, 2 mM MgCl2, 3 mM CaCl2, 10 mM glucose, and 10 mM HEPES at pH 7.4 (adjusted with KOH) and 300 mOsm. Whole-cell recordings were made under visual guidance using GFP-fluorescence to identify GFP^+^ cells, and were then performed using recording pipettes (approximately 3–5 MΩ) filled with an intracellular solution containing: 0.2 mM EGTA, 130 mM K-gluconate, 6 mM KCl, 3 mM NaCl, 10 mM HEPES, 4 mM ATP-Mg, 0.4 mM GTP-Na, 14 mM phosphocreatine-di(Tris) at pH 7.2 and 285 mOsm. Series and input resistance were measured in voltage-clamp with a 400 ms, 10 mV step from a -60 mV holding potential (filtered at 30 kHz and sampled at 50 kHz). The input resistance ranged from 50 to 1500 MΩ. Currents were filtered at 3 kHz, acquired and digitized at 10 kHz usingClampex10.3 software (Molecular Devices). Action potentials were recorded in current-clamp and elicited by a series of current injections ranging from -20 to 200 pA at 20 pA increments and 400 ms in duration. For all voltage-clamp recordings, cells were clamped at -60 mV except for the voltage step protocol. All current-clamp recordings were made at resting membrane potential or without any current injection. Sodium currents were recorded under voltage-clamp in response to a series of voltage steps ranging from -60 to 60 mV with 10 mV increments and 200 ms duration.

### Teratoma Generation

Briefly, around 5 million iPSCs were injected into testis capsules of immunocompromised NOD/SCID mice (aged 2–3 months). Tumor samples were collected in 8–12 weeks, fixed in 10% neutral buffered formalin (NBF), and processed for paraffin embedding and hematoxylin and eosin (H&E) staining. Experimental protocols were approved by the Institutional Animal Care and Use Committee at University of Texas Southwestern.

### Statistical Analysis

qRT-PCR assays for iPSC characterization were performed once, and all other experiments were performed in triplicate for at least three times. Data were presented as the mean ± SEM values. Statistical significance between groups was determined using two-tailed student’s *t*-test or two-way ANOVA by GraphPad Prism. The differences were considered significant when *p*-value was less than 0.05.

## Results and Discussion

### Generation of Human iPSCs

**FIGURE 1:**
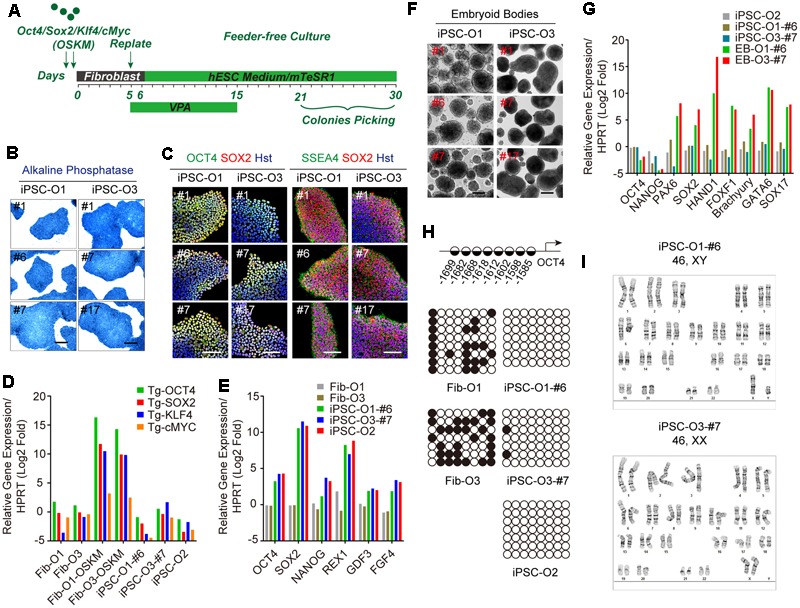
Generation and characterization of adult fibroblast-derived iPSCs. **(A)** Diagram of iPSC generation by Yamanaka factors. **(B)** AP staining of iPSC clones. Scale bars, 250 μm. **(C)** Immunocytochemical analysis of pluripotency markers OCT4, SOX2, and SSEA4 in iPSC clones. DNA was stained with Hoechst 33342. Scale bars, 100 μm. **(D)** qRT-PCR analysis of retroviral transgenes in iPSCs 5 days after virus transduction. “Tg” indicates primers detecting the transgene only. **(E)** qRT-PCR analysis of endogenous ESC markers in iPSCs. **(F)** EBs from iPSC clones in suspension culture at day 7. Scale bars, 200 μm. **(G)** qRT-PCR analysis of three germ layer markers in EBs at day 14. **(H)** Methylation state of the promoter region of OCT4. Positions of the CpG dinucleotides relative to the OCT4 transcription start site are provided. Open and closed circles indicate unmethylated and methylated CpGs, respectively. **(I)** Karyotypes of iPSCs.

To assess the aging status during iPSC reprogramming from fibroblasts, we obtained three lines of young fibroblasts (#Y1∼#Y3: aged 3 months, 2 years, and 3 years) and old fibroblasts (#O1∼#O3: aged 53 years, 55 years, and 71 years) respectively, from Coriell (**Table 1**). As four lines of their corresponding iPSCs were also obtained, we generated iPSCs from the other two lines of old fibroblasts (#O1 and #O3). To reprogram the fibroblasts, we introduced the four Yamanaka factors (OCT4, SOX2, KLF4, and c-MYC) using moloney-based retrovirus (**Figure 1A**). Three clones were successfully derived from each fibroblast line and displayed hallmarks of pluripotency as determined by AP staining as well as markers for pluripotency such as OCT4, SOX2, and SSEA4 (**Figures 1B,C**). qRT-PCR analysis using primers specific for retroviral transcripts revealed gradual silencing of all four retroviruses in established iPSCs (**Figure 1D**). Meanwhile, ESC markers such as OCT4, SOX2, NANOG, reduced expression 1 (REX1), fibroblast growth factor 4 (FGF4), and growth and differentiation factor 3 (GDF3) were dramatically induced (**Figure 1E**). *In vitro* differentiation of iPSCs in suspension gave rise to EBs (**Figure 1F**), which can be further differentiated into three germ layers, confirmed by qRT-PCR (**Figure 1G**). Bisulfite sequencing analysis demonstrated that the endogenous OCT4 promoters of established iPSCs were hypomethylated, whereas they were hypermethylated in fibroblast donors (**Figure 1H**). Moreover, the established iPSCs also retained normal karyotypes (**Figure 1I**), and formed teratomas, which included representative tissues originating from three germ layers, such as neural epithelium, cartilage, muscle and glandular structures (Supplementary Figure 1). We thus used these fully characterized iPSC clones in subsequent neural differentiation and aging analyses.

### iPSC Reprogramming Resets Aging-Associated Features

**FIGURE 2:**
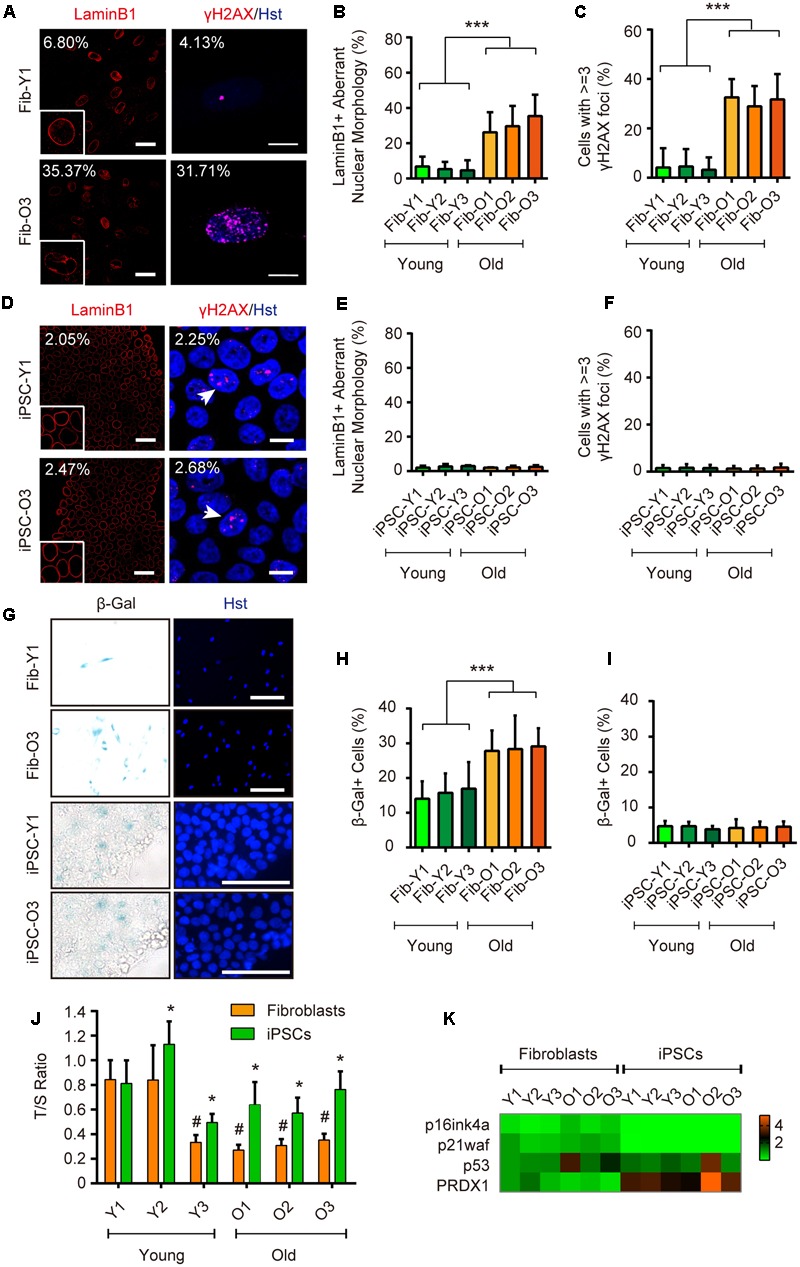
iPSC reprogramming resets aging-associated features. **(A–C)** Immunocytochemical analysis of LaminB1 and γH2AX in young and old fibroblasts. Scale bars, Lamin B1 50 μm; γH2AX 10 μm. ^∗∗∗^*p* < 0.001. **(D–F)** Immunocytochemical analysis of Lamin B1 and γH2AX in iPSCs derived from young and old donor fibroblast. Scale bars, Lamin B1 50 μm; γH2AX 10 μm. **(G–I)** SA-β-Gal staining of young and old fibroblasts, and their corresponding iPSCs. Scale bars, 100 μm. ^∗∗∗^*p* < 0.001. **(J)** Relative telomere lengths expressed as T/S ratios by qRT-PCR analysis in fibroblasts and iPSCs. ^∗^*p* < 0.05 fibroblasts compared to their corresponding iPSCs. ^#^*p* < 0.05 compared to the young fibroblast GM00041. **(K)** qRT-PCR analysis of gene expression in fibroblasts and iPSCs.

In order to address the aspects of cellular age during iPSC reprogramming, we then utilized a set of markers including those derived from Hutchinson-Gilford progeria syndrome (HGPS), which is a rare and fatal human premature aging disease (Scaffidi and Misteli, 2005). Immunostaining of Lamin B1 showed that old fibroblasts apparently displayed more aberrant nuclear morphology (such as folding and blebbing) (**Figures 2A,B**). DNA damage induces extensive double-strand breaks (DSBs), which can be visualized as γH2AX foci. In this study, those cells with greater than or equal to three γH2AX foci were counted as aged cells. Immunostaining of γH2AX showed that around 31.7% of cells possessed aged foci in old fibroblasts, compared to around 4.1% in young fibroblasts (**Figures 2A,C**). In contrast, the ratios of nuclear morphology abnormalities and DNA damage reduced to comparable minimal levels in reprogrammed iPSCs from both young and old donors (**Figures 2D–F**). The SA-β-Gal activity was also increased in old fibroblasts by approximately two-fold (29.1% for old fibroblasts versus 14.0% for young fibroblasts), whereas decreased to around 4.4% in average after reprogrammed to iPSCs (**Figures 2G–I**). Similarly, aging contributed to higher levels of mitochondrial superoxide in old fibroblasts, which were reverted in iPSCs (Supplementary Figure 2).

Telomere shortening occurs concomitant with organismal aging, and can be relatively measured by the T/S ratio (Cawthon, 2002). Compared to donor fibroblasts, iPSCs showed significantly increased telomere lengths indicated by a higher T/S ratio (**Figure 2J**). qRT-PCR analysis also revealed that iPSC reprogramming led to dramatically decreased expression of classical senescence-associated markers, such as p16ink4a and p21waf, whereas the expression of an antioxidant enzyme peroxiredoxin 1 (PRDX1), a strong regulator in delaying the onset of aging (Aeby et al., 2016), was greatly enhanced (**Figure 2K**).

**FIGURE 3:**
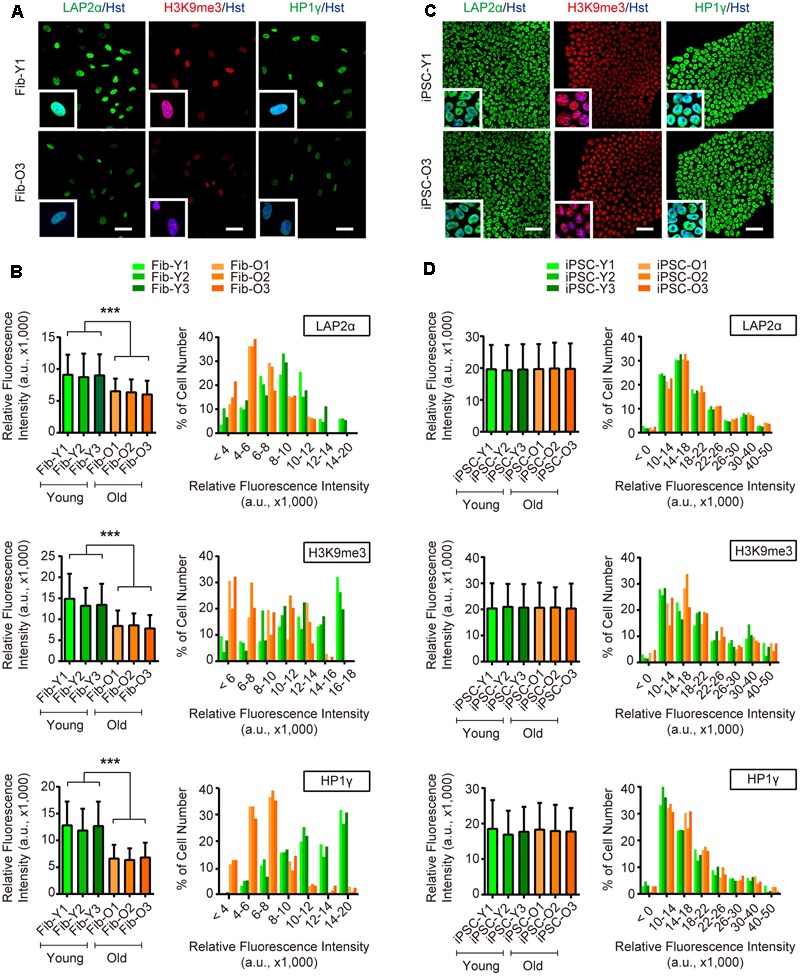
iPSC reprogramming reverts aging-associated changes on heterochromatin and nuclear organization. **(A,B)** Immunocytochemical analysis of H3K9me3, LAP2α, and HP1γ in young and old fibroblasts. Scale bars, 50 μm. ^∗∗∗^*p* < 0.001. **(C,D)** Immunocytochemical analysis of H3K9me3, LAP2α, and HP1γ in iPSCs derived from young and old donor fibroblasts. Scale bars, 50 μm. The data were plotted as average fluorescence intensity and distributions of relative fluorescence intensity from single cells. a.u., arbitrary units.

Long-term maintenance of nuclear architecture and chromosome stability is pivotal to normal cellular functions over a lifetime (Oberdoerffer and Sinclair, 2007). To investigate the nuclear and chromosome architectures, we assessed three previously reported markers (Miller et al., 2013), including nuclear lamina-associated protein 2α (LAP2α), heterochromatin markers trimethylated H3K9 (H3K9me3) and heterochromatin protein 1γ (HP1γ). Our analysis showed that aging in fibroblasts led to global reductions of LAP2α as well as H3K9me3 and HP1γ, suggesting an impairment of nuclear and chromosome stability during aging (**Figures 3A,B**). In sharp contrast, these makers were highly expressed in reprogrammed iPSCs and were indistinguishable between iPSCs derived from either young or old donors (**Figures 3C,D**). Taken together, our results indicate that iPSC reprogramming resets many aspects of aging-associated features from fibroblast donors.

### Generation of MNs from iPSCs and Fibroblasts

**FIGURE 4:**
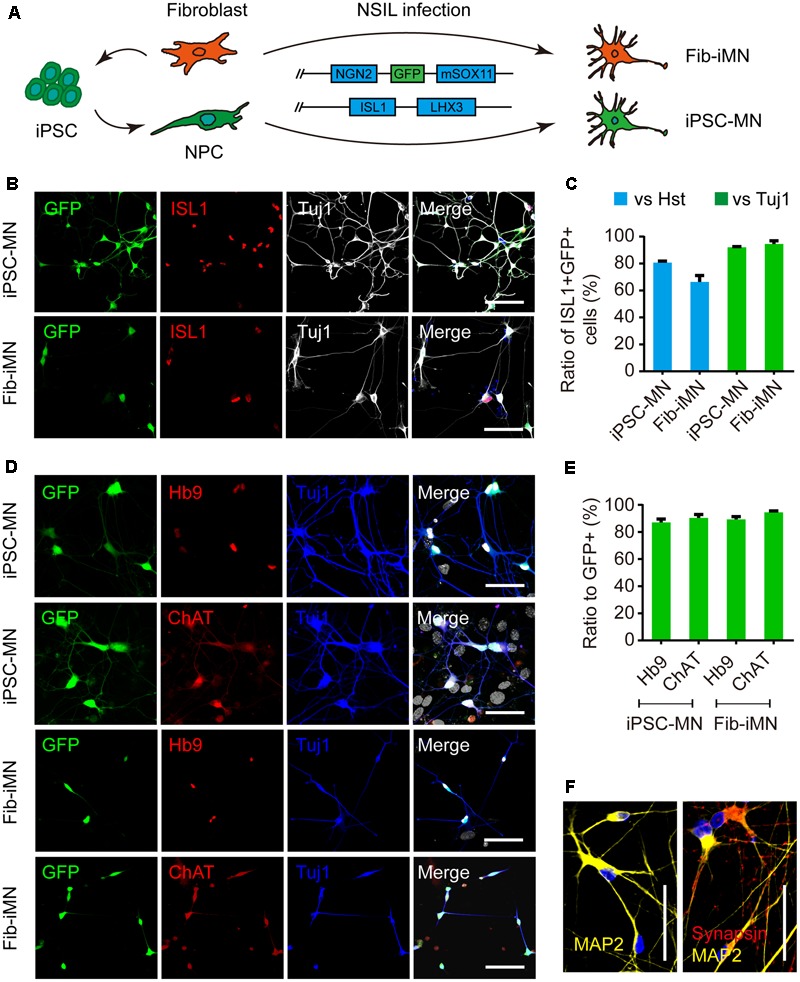
Generation of MNs from iPSCs and donor fibroblasts. **(A)** Diagram of MN generation by the NSIL method, which requires NGN2, SOX11, ISL1, and LHX3 in two lentiviruses. **(B,C)** Ratios of ISL1^+^GFP^+^ to Hoechst 33342 or Tuj1^+^ cells after the co-transduction of two lentiviruses at 5 dpi. Scale bars, 100 μm. **(D,E)** Immunostaining of MN markers (Hb9 and ChAT) and conversion efficiencies. Scale bars, 100 μm. **(F)** Immunostaining of MAP2 and Synapsin for iPSC-MNs. Scale bars, 50 μm.

Next, we focused our analysis on MNs, which are the principal neuronal subtype involved in human ALS. Multiple methods with variable efficiencies were previously used to differentiate iPSCs into MNs (Sances et al., 2016). However, none of these methods is applicable for direct reprogramming of donor fibroblasts to MNs. To minimize potential complications due to methodological variances, we established a protocol that could be employed to derive MNs both from iPSCs and directly from donor fibroblasts. This was to use the same set of four transcription factors termed NSIL (NGN2, SOX11, ISL1, and LHX3) to induce MNs from either donor fibroblasts (Liu et al., 2016) or iPSC-derived NPCs (Hester et al., 2011) (**Figure 4A**).

We first differentiated iPSCs toward NPCs through induction of the RA signaling pathway, according to previously reported methods (Yang et al., 2014) (Supplementary Figure 3A). We also incorporated into our analysis with four ALS patient-derived iPSC lines (two lines each with SOD1 or FUS mutation) (**Table 1**). All of the iPSC lines were efficiently induced to generate neurospheres (Supplementary Figure 3B) and yield highly pure NPCs. These NPCs were NESTIN/SOX2/PAX6 triple positive and also expressed polysialylated neuronal cell adhesion molecule (PSA-NCAM) and the proliferation marker Ki67 (Supplementary Figures 3C–E).

Spontaneous differentiation of the generated NPCs gave rise to both MAP2^+^ neurons and GFAP^+^ astrocytes (Supplementary Figure 3F). A subtype analysis of the differentiated neurons showed that the majority were glutamatergic (74.0% ± 1.3%) (Supplementary Figures 3G–I). HB9^+^ MNs on the other hand were rarely detected, which was also a reason for us to employ the NSIL method to induce MNs from NPCs.

The NSIL method uses two lentiviruses to express NGN2/SOX11/GFP and ISL1/LHX3, respectively. They showed to have 80.7% (iPSC-MN) or 66.4% (Fib-iMN) co-transduction as indicated by ISL1^+^GFP^+^/Hoechst 33342 ratios, and those ISL1^+^GFP^+^ cells constituted more than 90% among Tuj1^+^ populations in both groups. As such, a highly pure population of MNs was obtained after differential replating to remove a majority of non-converted cells (**Figures 4B,C**). Importantly, NSIL method generated HB9^+^ and ChAT^+^ MNs with comparable efficiencies from donor fibroblasts and iPSC-derived NPCs (iPSC-MNs: 87.0% ± 2.6% Hb9^+^/GFP^+^ and 90.4% ± 2.5% ChAT^+^/GFP^+^; Fib-iMNs: 94.5% ± 1.3% Hb9^+^/GFP^+^ and 94.5% ± 2.9% ChAT^+^/GFP^+^) (**Figures 4D,E**). Furthermore, those iPSC-MNs expressed mature neuronal marker MAP2 and the presynaptic marker synapsin I in a discrete punctate pattern (**Figure 4F**). These results indicate that MNs can be efficiently induced from adult fibroblasts as well as iPSC-derived NPCs.

Fib-iMNs are mature and suitable for modeling ALS-specific phenotypes (Liu et al., 2013, 2016). We then sought to validate the functionality of iPSC-MNs. Their electrophysiological properties were determined through whole-cell patch-clamp recordings. The recorded MNs fired repetitive action potentials upon current injection when examined at 35 dpi (Supplementary Figure 4A). In voltage-clamp mode, those MNs displayed inward and outward currents upon voltage steps, resembling sodium and potassium currents through voltage-gated sodium and potassium channels, respectively (Supplementary Figures 4B,C). Interestingly, under a stress condition caused by withdrawal of neurotrophic factors, MNs from ALS-iPSCs showed a significant reduction in soma size and survival rate when compared to those of healthy controls (Supplementary Figures 4D–F).

### Fib-iMNs Maintain Aging-Associated Features

**FIGURE 5:**
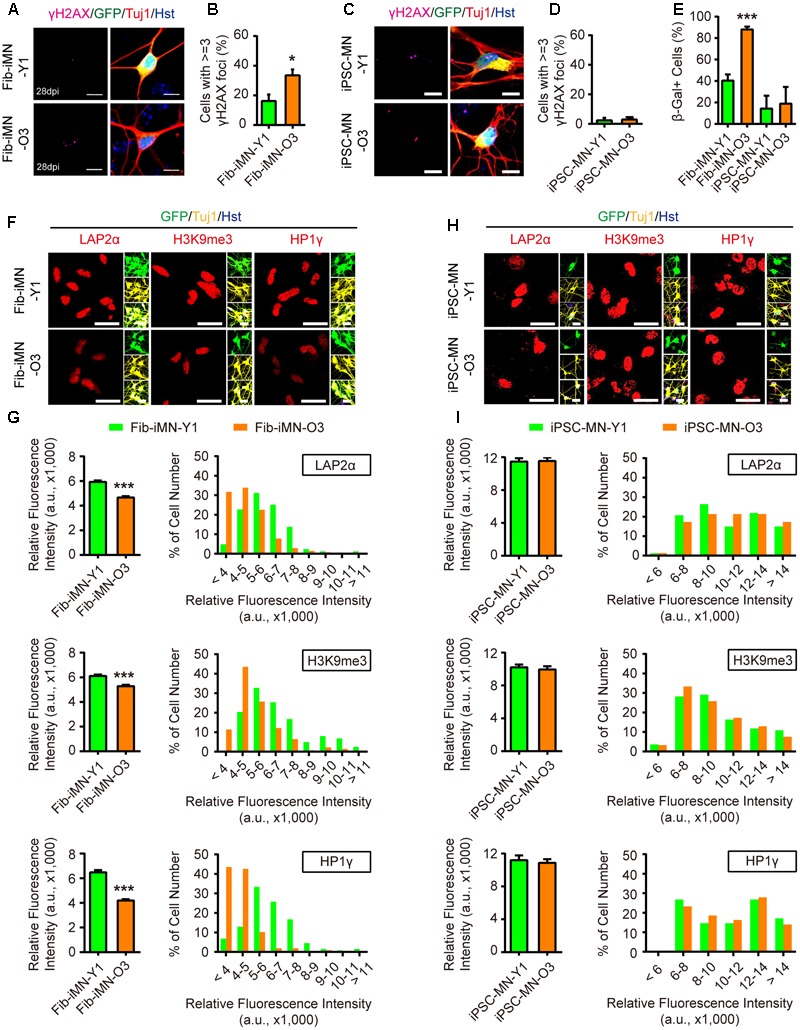
Directly reprogrammed Fib-iMNs maintain aging-associated features. **(A,B)** Age-dependent accumulation of γH2AX foci in Fib-iMNs. Scale bars, 10 μm. ^∗^*p* < 0.05. **(C,D)** Rare γH2AX foci in iPSC-MNs. Scale bars, 10 μm. **(E)** SA-β-Gal staining of MNs. ^∗∗∗^*p* < 0.001. **(F,G)** Confocal analysis of the indicated markers in Fib-iMNs. Scale bars, 25 μm. ^∗∗∗^*p* < 0.001. **(H,I)** Confocal analysis of the indicated markers in iPSC-MNs. Scale bars, 25 μm. The data were plotted as average fluorescence intensity and distributions of relative fluorescence intensity from single cells. a.u., arbitrary units.

These above data indicate that MNs can be efficiently generated from iPSCs or directly from donor fibroblasts. We then examined their aging status. When compared to Fib-iMNs from young donor, Fib-iMNs from old donor had a much higher number of cells containing γH2AX foci (33.5% in old Fib-iMNs vs. 16.2% in young Fib-iMNs) (**Figures 5A,B**). The old Fib-iMNs also had more than two-fold cells stained positive for SA-β-Gal (40.3% and 88.0% for young and old Fib-iMNs, respectively) (**Figure 5E**). This result recapitulates what were observed in their donor fibroblasts (**Figure 2H**). Concomitantly, confocal microscopy revealed that old Fib-iMNs had reduced levels of markers that are associated with heterochromatin and nuclear organization (**Figure 5F**). Quantification showed that there was an average reduction of 21.3%, 13.5% and 35.3% for LAP2α, H3K9me3, and HP1γ, respectively, in old than young Fib-iMNs (**Figure 5G**). Given the heterogeneous levels of these markers in individual Fib-iMNs, we also did a distribution analysis of cells with different staining intensity and found that a majority of old Fib-iMNs had lower levels of these markers (**Figure 5G**). Interestingly, the intensity distribution of H3K9me3 in old Fib-iMNs appeared less segregated by age than in aging fibroblasts (**Figures 3B, 5G**). This is probably due to a cell type specific role of H3K9me3 in fibroblasts versus neurons, or alternatively due to epigenetic remodeling during iMN conversion.

In sharp contrast, no any differences on aging-associated markers were detected between different iPSC-MNs. All these MNs showed low levels of γH2AX staining and SA-β-Gal activity (**Figures 5C–E**). The levels of LAP2α, H3K9me3, and HP1γ were also indistinguishable (**Figures 5H,I**). These results suggest that aging-associated features are not maintained in MNs once they pass through the iPSC stage. Taken together, our data reveal that directly reprogrammed Fib-iMNs rather than iPSC-MNs maintain the aging memories from their donor fibroblasts.

## Conclusion

**FIGURE 6:**
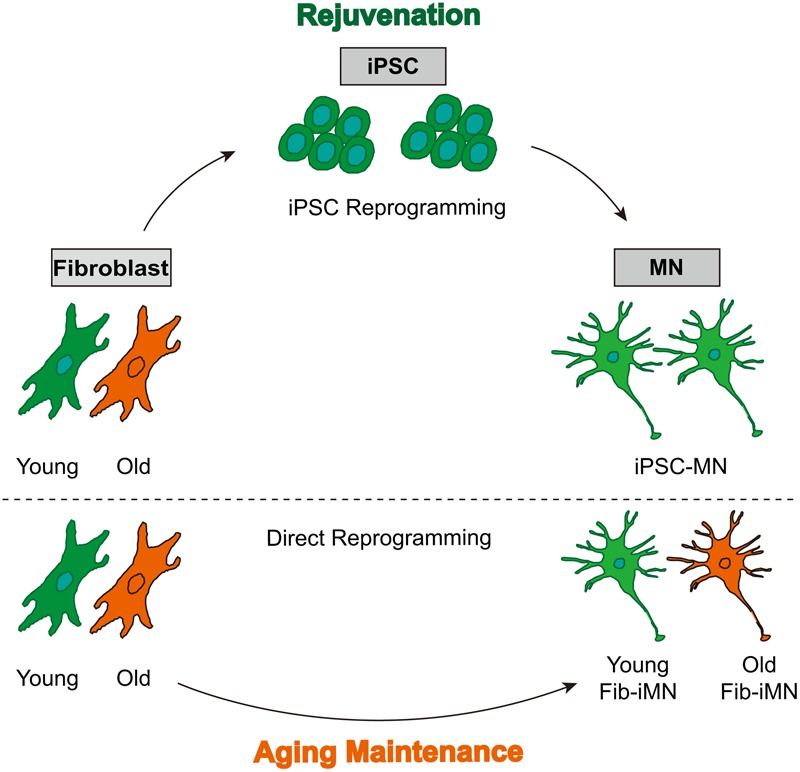
A model showing rejuvenation through reprogramming into pluripotency and aging maintenance via direct reprogramming. iPSC-MNs from old donor fibroblasts do not regain age-associated hallmarks, whereas directly reprogrammed Fib-iMNs maintain aging memories from old donors.

In this study, comparative analysis of many aging-associated features reveals a process of rejuvenation in iPSCs and their derived MNs, as well as aging maintenance in directly reprogrammed Fib-iMNs (**Figure 6**). Such features of Fib-iMNs may render them uniquely suitable for modeling late-onset hallmarks of MNDs such as ALS. Our results on spinal cord Fib-iMNs are in general agreement with previous studies showing that aging features are retained in directly reprogrammed brain neurons (Mertens et al., 2015; Huh et al., 2016). Nonetheless, we systematically analyzed a list of aging hallmarks that were not examined in these previous studies. Most importantly, we took advantage of our NSIL method for robust generation of MNs either directly from adult human fibroblasts or from fibroblast-derived iPSCs. Direct comparisons between these MNs allowed us to reveal the impact of different reprogramming methods on aging features.

In light of aging preservation in directly reprogrammed neurons, they may show aging-associated phenotypes that are not accessible in iPSC-derived neurons. For example, Mertens et al. revealed that directly reprogrammed neurons display age-specific transcriptional profiles and the loss of a nuclear transport receptor and nucleocytoplasmic compartmentalization. These features were not observed in iPSC-derived rejuvenated neurons (Mertens et al., 2015). Similarly, directly reprogrammed glutamatergic neurons from C9orf72 ALS fibroblasts showed dipeptide repeats-induced impairments in nucleocytoplasmic transport (Jovicic et al., 2015). It should be very interesting in the future to examine neuron subtype-specific and potentially disease-relevant phenotypes in the context of aging.

Due to rejuvenation of neurons from iPSCs, our results also raise concerns on using iPSCs to model age-dependent neurodegenerations. Such concerns have already drawn attention, as strategies are emerging to induce aging-like features in iPSC-derived cells, for example, by telomerase manipulation (Vera et al., 2016), progerin treatment (Miller et al., 2013), or prolonged culture (Petrini et al., 2017). Clearly, a cell culture system maintaining aging features such as our directly reprogrammed MNs will greatly facilitate our understanding of neurodegeneration and therapeutic identification and validation. Furthermore, future in-depth analysis on aging and neuropathology through genomics and proteomics approach is clearly warranted.

## Ethics Statement

All human cell lines in this study were from commercial sources or gifts provided by the other lab.

## Author Contributions

YT, M-LL, and C-LZ conceived and designed the experiments. YT performed all experiments related to human iPSCs and molecular biology. M-LL carried out direct reprogramming and related experiments. TZ conducted experiments related to electrophysiology. YT and C-LZ prepared the manuscript. All authors reviewed and approved the final manuscript.

## Conflict of Interest Statement

The authors declare that the research was conducted in the absence of any commercial or financial relationships that could be construed as a potential conflict of interest.
