# Supplementary material for: Direct Reprogramming Rather than iPSC-Based Reprogramming Maintains Aging Hallmarks in Human Motor Neurons
Source: Front Mol Neurosci. 2017 Nov 2;10:359. doi: 10.3389/fnmol.2017.00359 (PMC5676779; doi:10.3389/fnmol.2017.00359)
Supplement: Supplementary file 1 [file Table_1.PDF]

Supplementary File 1. List of primers used in this study.

Genes	Forward Primer
	**Reverse Primer**
	**Quantitative Real-time PCR**
Tg-OCT4	CCCCAGGGCCCCATTTTGGTACC
	TTATCGTCGACCACTGTGCTGCTG
Tg-SOX2	GGCACCCCTGGCATGGCTCTTGGCTC
	TTATCGTCGACCACTGTGCTGCTG
Tg-KLF4	ACGATCGTGGCCCCGGAAAAGGACC
	TTATCGTCGACCACTGTGCTGCTG
Tg-cMYC	CAACCGAAAATGCACCAGCCCCAG
	TTATCGTCGACCACTGTGCTGCTG
OCT4	GACAGGGGGAGGGGAGGAGCTAGG
	CTTCCCTCCAACCAGTTGCCCCAAAC
SOX2	GGGAAATGGGAGGGGTGCAAAAGAGG
	TTGCGTGAGTGTGGATGGGATTGGTG
NANOG	CAGCCCCGATTCTTCCACCAGTCCC
	CGGAAGATTCCCAGTCGGGTTCACC
REX1	CAGATCCTAAACAGCTCGCAGAAT
	GCGTACGCAAATTAA AGTCCAGA
GDF3	CTTATGCTACGTAAAGGAGCTGGG
	GTGCCAACCCAGGTCCCGGAAGTT
FGF4	CTACAACGCCTACGAGTCCTACA
	GTTGCACCAGAAAAGTCAGAGTTG
PAX6	GCCCTCACAAACACCTACAG
	TCATAACTCCGCCCATTCAC
SOX1	GCGGAGCTCGTCGCATT
	GCGGTAACAACTACAAAAAACTTGTAA
HAND1	TCCCTTTTCCGCTTGCTCTC
	CATCGCCTACCTGATGGACG
FOXF1	AAAGGAGCCACGAAGCAAGC
	AGGCTGAAGCGAAGGAAGAGG
Brachyury	AATTGGTCCAGCCTTGGAAT
	CGTTGCTCACAGACCACA
GATA6	TGTGCGTTCATGGAGAAGATCA
	TTTGATAAGAGACCTCATGAACCGACT
SOX17	TTCGTGTCCAAGCCTGAGATG
	GTCGGACACCACCGAGGAA
P16ink4a	CCAGCACCGGAGGAAGAAAG

	ATCATGACCTGGATCGGCCT
P21waf	TCACTGTCTTGTACCCTTGTGC
	GGCGTTTGGAGTGGTAGAAA
P53	GCTCACTCCAGCCACCTGAA
	GCCCTTCTGTCTTGAACATGAGTT
PRDX1	TTGTTCTTGCCTGGTGTCGG
	CTTCCTATCAGCTTGGGGTTCA
HPRT	GCTTTCCTTGGTCAGGCAGTA
	GTCTGGCTTATATCCAACACTTCGT
	**Telo-PCR**
TELO	CGGTTTGTTTGGGTTTGGGTTTGGGTTTGGGTTTGGGTT
	GGCTTGCCTTACCCTTACCCTTACCCTTACCCTTACCCT
36B4	CAGCAAGTGGGAAGGTGTAATCC
	CCCATTCTATCATCAACGGGTACAA
β-GLOBIN	GCTTCTGACACAACTGTGTTCACTAGC
	CACCAACTTCATCCACGTTCACC
	**Bisulfite Sequencing**
bs-OCT4	AATAGATTTTGAAGGGGAGTTTAGG
	TTCCTCCTTCCTCTAAAAAACTCA
