# Supplementary material for: Direct Reprogramming Rather than iPSC-Based Reprogramming Maintains Aging Hallmarks in Human Motor Neurons
Source: Front Mol Neurosci. 2017 Nov 2;10:359. doi: 10.3389/fnmol.2017.00359 (PMC5676779; doi:10.3389/fnmol.2017.00359)
Supplement: Supplementary file 2 [file Data_Sheet_1.pdf]

Supplementary Figures

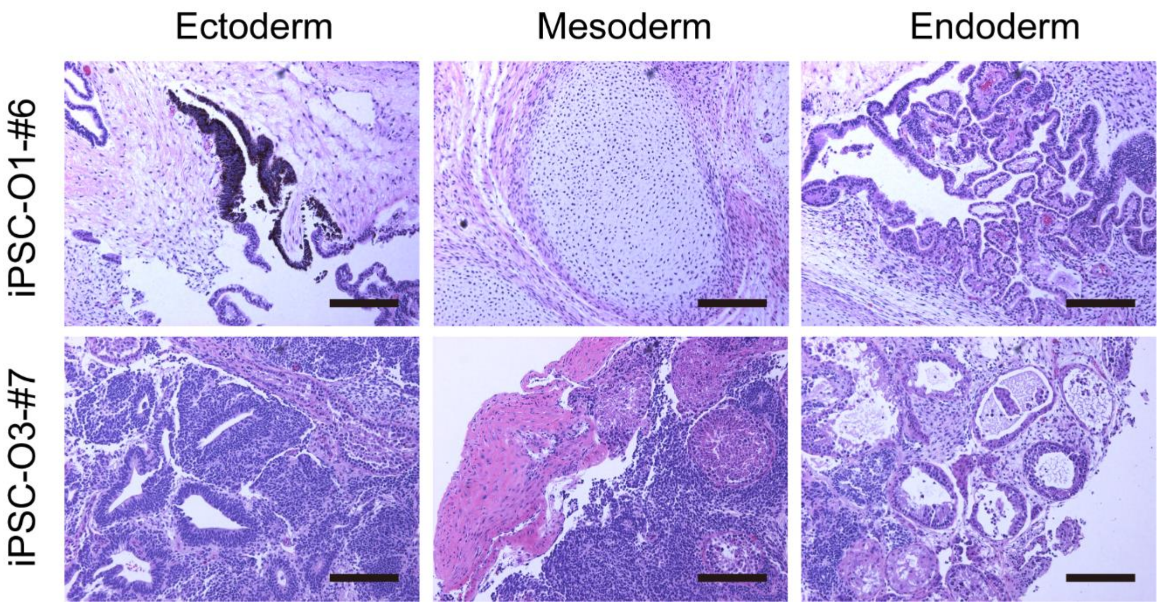

Supplementary Figure 1. H&E staining of teratoma sections showed differentiation of iPSCs to various tissues of three germ layers, such as neural epithelium, cartilage, muscle and glandular structures. Scale bars, 250 μm.

Supplementary Figures

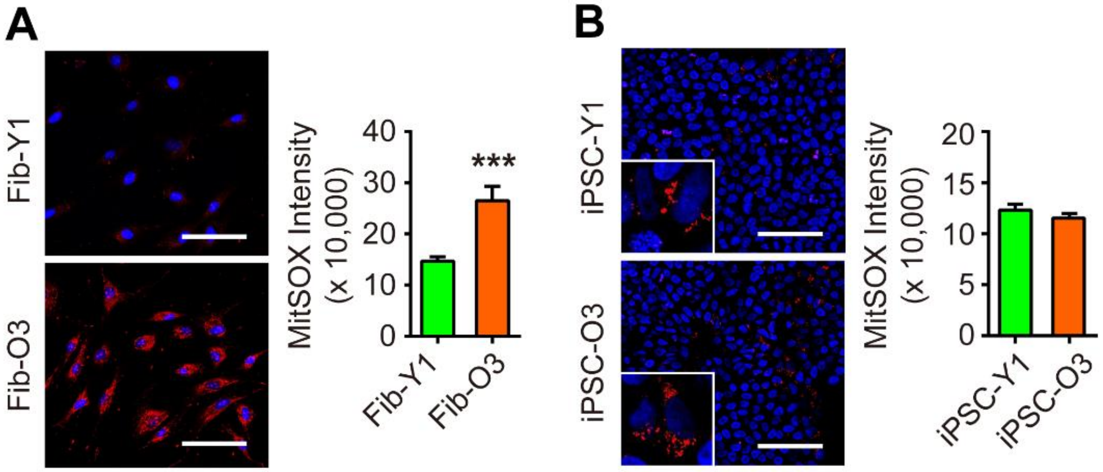

Supplementary Figure 2. Analysis of mitochondrial superoxide in young and old fibroblasts **(A)**, as well as their corresponding iPSCs **(B)**. Scale bars, 100 µm. ***p<0.001.

Supplementary Figures

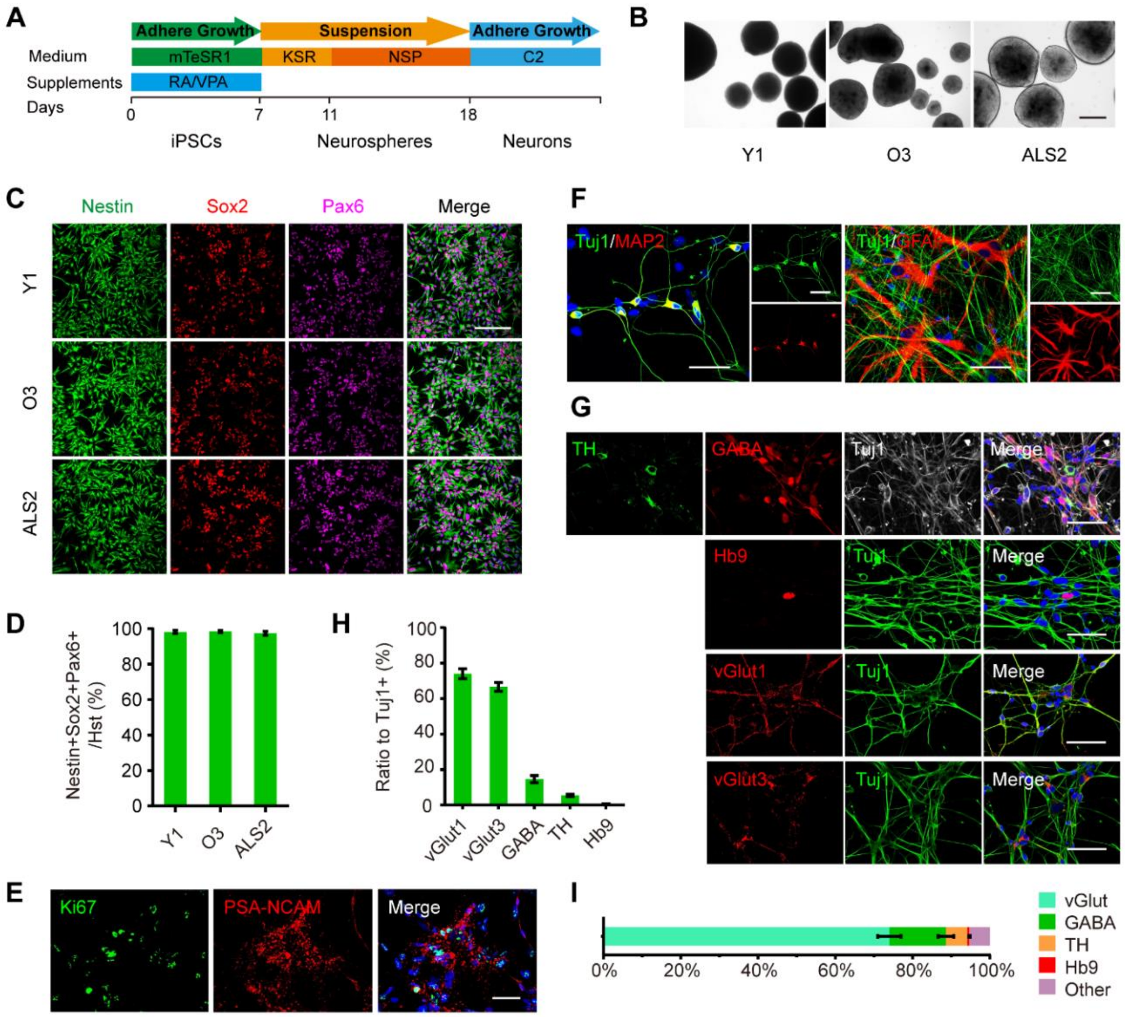

Supplementary Figure 3. NPC generation from iPSCs and neural differentiation. **(A)** Diagram of NPC generation from iPSCs. KSR: KSR medium; NSP: Neurosphere medium; C2: C2 medium. **(B)** Neurosphere formation at day 18. Scale bar, 200 µm. **(C, D)** Immunostaining of NPC markers (Nestin, Sox2 and Pax6). Scale bar, 200 µm. **(E)** Immunostaining of Ki67 and PSA-NCAM. Scale bar, 50 µm. **(F)** NPCs were spontaneously differentiated into neurons and astrocytes, represented by Tuj1/MAP2 staining and GFAP staining, respectively. Scale bars, 50 µm. **(G-I)** Neural subtypes of spontaneously differentiated neurons. Scale bars, 50 µm.

Supplementary Figures

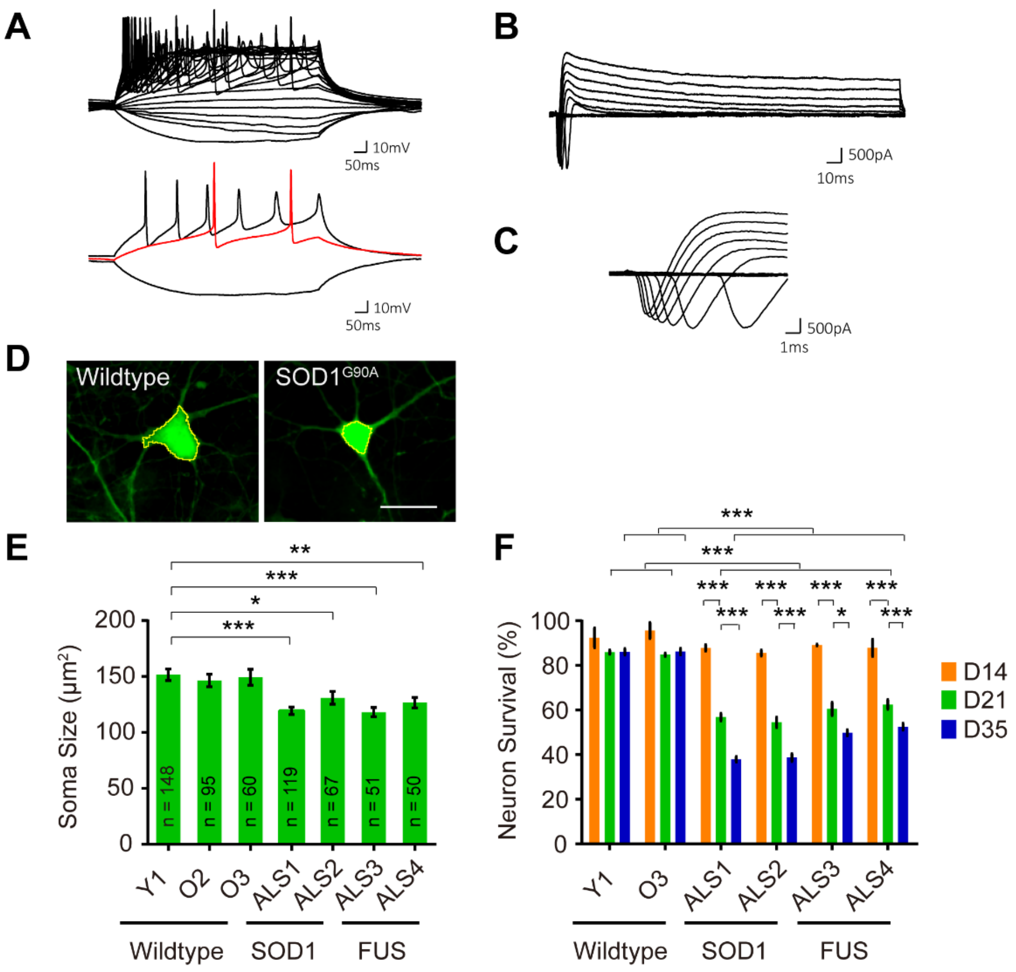

Supplementary Figure 4. Characterization of iPSC-MNs from healthy and ALS donors. **(A)** Repetitive action potential waveforms recorded under current-clamp mode for the SOD1^{G90A}-iPSC derived MNs. The precondition sweep, the sweep immediately above threshold (in red), and the sweep at the highest firing frequency are shown in the lower panel. **(B)** Representative waveform of inward and outward currents upon voltage steps is shown. **(C)** A zoomed-in view of inward currents, resembling sodium currents, presented in (**B**) is shown. **(D)** Representative images of healthy and SOD1^{G90A}-iPSC derived MNs. Healthy: iPSC-O3; SOD1^{G90A}: iPSC-ALS2. Scale bar, 20 μm. **(E)** Soma size (μm^2) of healthy and diseased iPSC derived MNs. *p<0.05; ***p<0.001. (**F**) Survival of healthy and diseased iPSC-MNs. *p<0.05; ***p<0.001.
